# THE DESIGN AND PILOTING OF A NEW GUIDE FOR ADDRESSING RESIDENT GOALS, PREFERENCES, AND PRIORITIES

**DOI:** 10.1093/geroni/igae098.1215

**Published:** 2024-12-31

**Authors:** Laci Cornelison, Mary Lou Ciolfi

**Affiliations:** Kansas State University, Manhattan, Kansas, United States; University of Maine Center on Aging, Bangor, Maine, United States

## Abstract

Engaging nursing home residents and their designated representatives in meaningful conversation about their needs and preferences for care, services, and environments is essential to high quality long-term care, as confirmed in the 2022 National Academies of Sciences, Engineering, and Medicine (NASEM) report. The Moving Forward Coalition’s Goal One Committee has created a quality improvement Guide for Addressing Resident Goals, Preferences, and Priorities (GPPs) to support nursing home care planning staff in improving and deepening care planning conversations with residents. The Guide includes key suggestions, tools, resources, and training material that will help homes lay a foundation for resident and family inclusion and empowerment in disclosing goals, preferences, and priorities (GPPs) and also in implementing those GPPs at the outset of a resident’s admission to care and as needs and preferences change over time. Goal Committee One leads will present the Guide purpose and design along with a review of included resources; review the feedback and suggestions from myriad nursing home stakeholders, including current nursing home residents, nursing home providers, and nursing home quality improvement organizations; outline the results of the Guide pilot in several nursing homes, and the upcoming planned Guide evaluation and wider dissemination.

